# Complex of Defense Polypeptides of Wheatgrass (*Elytrigia elongata*) Associated with Plant Immunity to Biotic and Abiotic Stress Factors

**DOI:** 10.3390/plants13172459

**Published:** 2024-09-03

**Authors:** Anna S. Barashkova, Alexey N. Smirnov, Eugene A. Rogozhin

**Affiliations:** 1 Shemyakin and Ovchinnikov Institute of Bioorganic Chemistry, Russian Academy of Sciences, 117997 Moscow, Russia; rea21@list.ru; 2 All-Russian Institute of Plant Protection, 196608 Saint Petersburg, Russia; 3 Department of Plant Protection, Institute of Agrobiotechnology, Timiryazev Russian State Agrarian University, 127550 Moscow, Russia; smirnov@timacad.ru; 4 Papanin Institute for Biology of Inland Waters, Russian Academy of Sciences, 152742 Borok, Russia

**Keywords:** defense peptides, biotic and abiotic stresses, structure analysis, antifungal activity, reactivating effect

## Abstract

Plant defense polypeptides play a crucial role in providing plants with constitutive immunity against various biotic and abiotic stressors. In this study, we explored a complex of proteins from wheatgrass (*Elytrigia elongata*) spikelets to estimate their role in the plant’s tolerance to various environmental factors. The current research shows that in vitro protein extracts from *E. elongata* spikelets possess antifungal activity against certain *Fusarium* species, which are specific cereal pathogens, at concentrations of 1–2 mg/mL. In this study, we reproduced these antifungal activities using a 4 mg/mL extract in artificial fungal infection experiments on wheat grain (*Triticum aestivum*) under controlled laboratory conditions. Furthermore, the tested extract demonstrated a protective effect on *Saccharomyces cerevisiae* exposed to hyper-salinity stress at a concentration of 2 mg/mL. A combined scheme of fractionation and structural identification was applied for the estimation of the diversity of defense polypeptides. Defensins, lipid-transfer proteins, hydrolase inhibitors (cereal bifunctional trypsin/alpha-amylase inhibitors from a Bowman–Birk trypsin inhibitor), and high-molecular-weight disease resistance proteins were isolated from the extract. Thus, wheatgrass spikelets appear to be a reservoir of defense polypeptides. Our findings contribute to a deeper understanding of plant defense proteins and peptides and their involvement in the adaptation to various stress factors, and they reveal the regulatory effect at the ecosystem level.

## 1. Introduction

Plants represent a fundamental part of terrestrial ecosystems. They provide food chain function, carbon sequestration, and global cycles of essential elements [1,2]. They face numerous stress factors throughout their ontogenesis. Some ecosystems challenge them with a composition of harsh conditions, such as high salinity, drought, and high insolation. Throughout their evolutionary history, plants have developed a number of defense mechanisms. Proteins and peptides are components of plants’ constitutive innate immunity and represent the first line of defense against various environmental stress factors. Therefore, they are of significant interest for fundamental and applied studies due to their role in responses to various stresses [3,4,5]. To date, a considerable number of antimicrobial peptides (AMPs) have been isolated from plants and predicted via genomic and transcriptomic data [6,7]. Plant AMPs are classified into the following eight families according to their structures: thionins, defensins, lipid-transfer proteins (LTPs), hairpin-like peptides, hevein-like peptides, snakins, knottins, and cyclotides [8]. The most well-studied and characteristic antimicrobial activity is implemented through pathogen membrane disruption; this mode of action is most characteristic of thionins and cyclotides and is less characteristic of LTPs and defensins [9,10]. In addition to the direct disruptive antimicrobial activity, plant AMPs have specific activities. Knottins and some hairpin-like peptides (from *Fagopyrum esculentum* and *Cucurbita maxima*) represent a wide group of enzyme inhibitors [11,12], which also include ribosome-inactivating peptides [13,14]. Defensins are known to block ion channels [15,16]. Cyclotides possess insecticidal and nematocidal activities [17,18]. Moreover, AMPs have been shown to participate in physiological processes in plants and are also involved in abiotic stress responses [19,20]. AMPs from plants are also found to be involved in contributions to the induction of plant immunity via the modulation of defense responses associated with molecular pattern- or effector-triggered immunity [3,21]. The defensins HsAFP1, RsAFP2, PvD1, and NaD1 are involved in the oxidative burst response to fungal infections [22,23]. Some of the peptide gene expression is regulated by phytohormones: the *SNAKIN2* gene is upregulated by abscisic and downregulated by gibberellins [24]. The defensin genes in *Arabidopsis thaliana* leaves [25] and the hevein-like peptides in *Wasabia japonica* are induced and regulated by jasmonate [26]. Based on their structures, some plant AMPs demonstrate cytotoxic properties that may potentially be applied in medicine and veterinary science [27].

Wild cereals (*Poaceae*) have adapted to the full range of environmental extremes experienced by plants. Their immune response to stress factors, compared with that of non-cultivated dicots, makes them interesting objects to study [28,29]. It has been shown that cereals produce a wide variety of defense proteins and peptides. Some of them are protective factors against abiotic stresses [30,31]. Hydrolase inhibitors, which are molecular-specific tools that provide cereals with immunity to insects, constitute a large proportion of the defense molecules; these molecules also include chitin-binding and lectin-like proteins [32,33,34,35]. Some of these inhibitors also possess antimicrobial properties [36,37]. Finally, there is a large group of antimicrobial peptides (AMPs) with various activities, including antifungal activities (defensins, thionins, lipid-transfer proteins, and hevein-like peptides) [38]. However, the deciphering of the *Poaceae* defense mechanisms remains relevant.

Most studies devoted to plant AMPs are focused on the structures and activities of individual molecules [39,40]. At the same time, the complex contribution of AMPs to the plant’s stress response is studied at the transcriptional level. In this study, we investigate a complex of polypeptides from *Elytrigia elongata* (Host) Nevski mature spikelets with defense functions that determine the effectiveness of the initial stage of plant ontogeny. *E. elongata* is distributed in relatively warm climates, including areas in the southern part of Eastern Europe, the Caucasus, the Mediterranean, Central Asia, and Iran. It mainly thrives in the Solonchak steppe grasslands, in salt marshes, along coastal regions, and in nearby areas. It is a good fodder plant. *E. elongata* is known to be resistant to fungal diseases and insect pests, low temperatures, and drought. Moreover, it is used in common wheat (*Triticum aestivum*) breeding [41,42,43]. *E. elongata* was chosen for this study because of its remarkable ecology and stress tolerance.

## 2. Results

### 2.1. Isolation of Protein Extract from Elytrigia elongata Spikelets

The proteins and peptides were obtained using a technique based on acetic acid extraction, followed by precipitation with ice-cold acetone. The total *E. elongata* spikelet protein extract (EePE) was desalted by solid-phase extraction (SPE). The EePE yield was 24 mg per gram of plant material. This exceeds the analogous indicator for wild cereal barnyard grass (*Echinochloa crusgalli*), which is a rich source of defense peptides belonging to various structural families [44]. First, EePE was tested for its biological activity.

### 2.2. Biological Activity of PE from E. elongata Spikelets

#### 2.2.1. Antifungal Action

To implement the “from function to structure” approach, we decided to evaluate the functional effect of EePE. Frequently, biological activity is more pronounced when a number of molecules of the same chemical nature, such as polypeptides, act together [45,46,47]. To assess the involvement of the protein and peptide components in the plant’s constitutive immune response, several tests were carried out. First, the growth suppression effect of EePE on filamentous fungi was evaluated. *Bipolaris sorokiniana*, *Fusarium graminearum*, *Fusarium culmorum*, and *Aspergillus niger*, the cereal root rot complex-forming fungi, were selected as test objects. Growth inhibition was estimated on solid media according to the occurrence of a growth suppression zone. The growth suppression effect was found only on *Fusarium* species at an EePE concentration higher than 1 mg/mL (Table 1).

**Figure 1:**
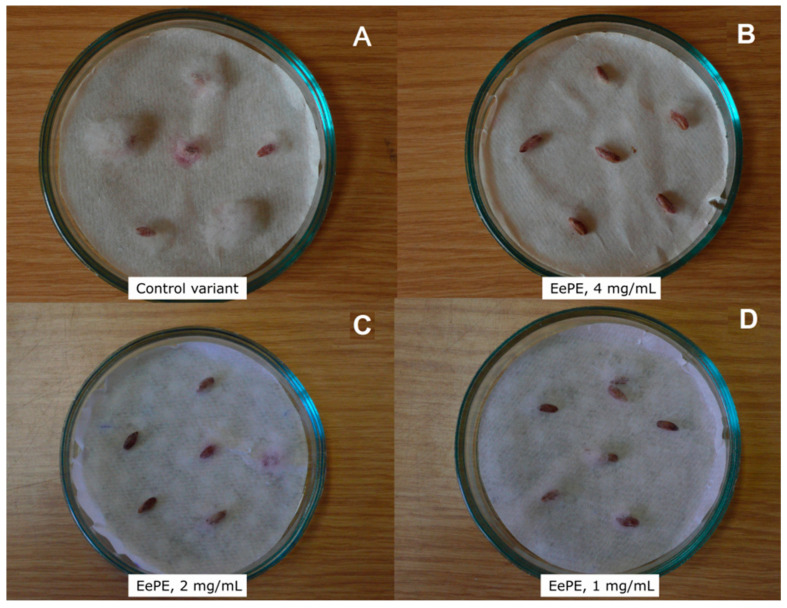
Development of *F. graminearum* applied to the wheat grain (*T. aestivum*) surfaces pretreated with *E. elongata* protein–peptide extract (EePE) after 72 h of incubation: (**A**)—control variant (70% aqueous ethanol); (**B**)—concentration of the EePE, 4 mg/mL (fungal development composed of “++”); (**C**)—concentration of the EePE, 2 mg/mL (fungal development composed of “+”); (**D**)—concentration of the EePE, 1 mg/mL (fungal development composed of “++”).

The effectiveness of the EePE from *E. elongata* was also examined on wheat (*T. aestivum*) grain. Briefly, the wheat grain was pretreated with EePE, then inoculated with one susceptible phytopathogenic fungus, *F. graminearum* (Figure 1). Initially, it was planned for this experiment to be carried out under natural conditions. However, the wheat grain batches had an extremely low degree of seed contamination. Consequently, the study parameters were changed in favor of an artificial background. It is worth noting that experimental batches of untreated grain may have a rather high baseline level of contamination with fungi as well as bacteria. This obfuscates the interpretation of test results and blurs the effect of the antimicrobial tested. The EePEs demonstrated a protective effect at the highest concentration tested—4 mg/mL—where fungal development did not exceed 20%, which corresponds to “++” in the mark scale (Figure 1B). In comparison with the control variant, the effect manifested by *F. graminearum* growth suppression on pretreated grain (Figure 1A) was retained over time. However, when treated at half and quarter concentrations—2 and 1 mg/mL—the protective effect was less pronounced but still distinguishable (Figure 1C,D).

#### 2.2.2. Protective Activity

To date, a considerable amount of experimental data on exometabolites of different species has been accumulated. It has been shown that microorganisms of various physiological groups release micro- or nanomols of substances to the environment during growth. These compounds have a stress-protective effect on prokaryotic and eukaryotic cells. It is believed that they act as signaling molecules. Typically, the activity of these metabolites is not species-specific, which leads to cross-acting between representatives of different taxa, including plants. In the next stage of the research, the EePE was tested for the presence of regulatory protective factors that could contribute to the survival of the microbial community under the hyper-influence of an abiotic stress factor (high salinity in the case of *E. elongata*).

To examine EePE protective potency, the yeast model was applied. Briefly, yeast cells (*Saccharomyces cerevisiae*) were preincubated with 2 mg/mL of EePE solution, whereupon the stress factor—bile salts, 2 g/L—was applied. The cells incubated without bile salts or without EePE pretreatment were considered as positive and negative controls, respectively. It was found that the average number of *S. cerevisiae* cells in the presence of the stress factor was 0.10 × 10^6^ (or 0.071% of their initial quantity). However, in the preincubated EePE, the average cell number of *S. cerevisiae* increased by up to 60% (0.16 × 10^6^ ± 7.5 × 10^3^). It is worth noting that the application of EePE at a concentration of 8 mg/L did not result in significant changes in the cells’ protective effect.

### 2.3. Isolation and Structure Analysis of Individual Polypeptides from E. elongata Spikelets

**Figure 2:**
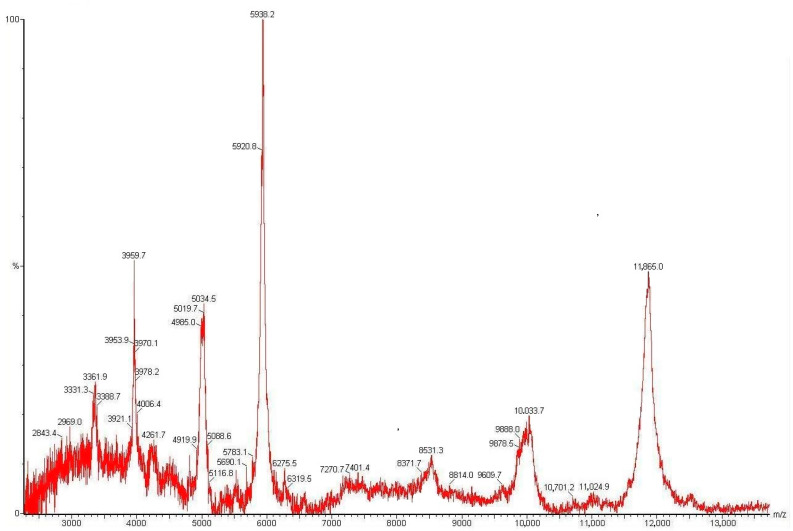
MALDI-TOF MS analysis of the total protein extract from *E. elongata* spikelets. Intense signals were obtained in the range of 3.3–6.0 kDa and individual signals in the range of 8.5–12.0 kDa. This indicates the presence of polypeptides. All *m*/*z* values are measured in average mode.

The explanation of the biological test results required fractionation of the total extract in order to identify the active ingredients. We focused on a search of the proteins and peptides involved in the plant’s molecular response to biotic and abiotic stress factors. First of all, we performed MALDI-TOFMS analysis of the EePE within the range of 1.0–20.0 kDa (Figure 2). The total mass spectrum showed several intense *m*/*z* signals in the range of 3.3–6.0 kDa and individual signals in the range of 8.5–12.0 kDa. The presence of polypeptides in a broad range of molecular masses suggests a diverse distribution of peptides at a qualitative level, which is typical for both wild [6] and cultivated cereals [48,49].

**Figure 3:**
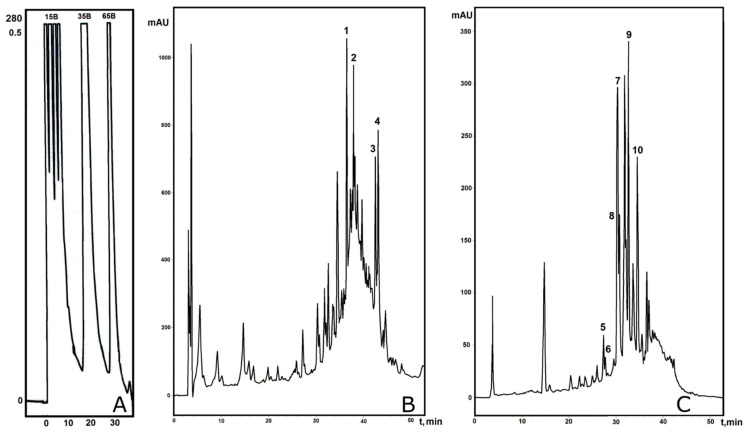
HPLC of fractions obtained after affinity chromatography: (**A**)—stepwise semi-preparative HPLC of fraction I eluted with 100 mM NaCl (for fraction II eluted with 500 mM NaCl, the profile looks quite similar); (**B**)—analytical RP-HPLC of subfraction Ee100-35B after stepwise RP-HPLC of fraction I; (**C**)—analytical RP-HPLC of subfraction Ee500-65B after stepwise RP-HPLC of fraction II. Peaks indicated by ciphers were collected manually and identified; numbering corresponds to that in Table 2.

The obtained data allowed us to devise a liquid chromatography-based fractionation strategy for the PE from *E. elongata* spikelets. To isolate individual polypeptides, we employed the well-proven fractionation scheme, which has previously been successfully applied to identify particular antimicrobial peptides from cereals [48,50,51]. Briefly, medium-pressure affinity chromatography on a Heparin HiTrap Sepharose column yielded two total fractions: fraction I, eluted with 100 mM NaCl, and fraction II, eluted with 500 mM NaCl (the profile is not shown). The SPE-desalted fractions were tested for antifungal properties against the most susceptible *F. graminearum*, at the same active concentration as that of the EePE previously tested in vitro. Growth suppression was observed in both fractions; the more pronounced suppression zone was in fraction II. Subsequently, both fractions were subjected to analytical reversed-phase HPLC (RP-HPLC). However, the resulting chromatographic profiles had low resolution; this was presumably due to the presence of storage proteins. Interestingly, pre-stage, medium-pressure gel chromatography did not improve the separation pattern. To improve the separation quality, the non-desalted fractions I and II were additionally separated by semi-preparative RP-HPLC in a stepwise gradient of 80% acetonitrile in 0.1% trifluoracetic acid (solvent B). As a result, three subfractions eluted with 15, 35, and 65% solvent B were collected for fractions I (Ee100-15B, Ee100-35B and Ee100-65B) and II (Ee500-15B, Ee500-35B and Ee500-65B), separately (Figure 3A). For all the subfractions, tests for antifungal activity against *F. graminearum* were carried out. The subfractions Ee100-35B and Ee500-65B demonstrated the highest growth inhibition effects at concentrations of 0.25 mg/mL and higher. Then, both subfractions were re-chromatographed by analytical RP-HPLC to obtain better resolution (Figure 3B,C).

The efficiency of the fractionation increased, and several compounds were eluted and subsequently collected manually for MALDI-TOF MS and Edman sequencing analysis. As a result, four defense polypeptides were identified from fraction I (100 mM NaCl) (Figure 3B and Table 2): two homologous Ee-LTP1 and Ee-LTP2 proteins, which share homology with non-specific lipid-transfer protein GPI-anchored 14-like from timothy grass (*Trifolium pratense*) (GenBank ID: XP_045800415.1); Ee-BFTI, a bifunctional trypsin/alpha-amylase inhibitor from cereals (alpha-amylase/trypsin inhibitor-like from panic grass (*Panicum virgatum*), GenBank ID: XP_039798854.1); and Ee-D5, a homologue of gamma-1-purothionin from wheat (*Triticum aestivum*) (UniProt/SwissProt ID: P20158.1). Fraction II (500 mM NaCl) demonstrated a high amount of peptide and protein components (Figure 3C and Table 2). Six AMPs were identified; three of them (Ee-D1, Ee-D2, and Ee-D6) were homologous to gamma-1-purothionin and gamma-2-purothionin from wheat (*Triticum aestivum*) (UniProt/SwissProt IDs: P20158.1 and P20159.1). According to the results of the BLASTP search, the second pair of AMPs (Ee-D3 and Ee-D4) possessed broader homology among the cereal defensins: defensin-like peptides from millet (*P. halli*) (GenBank ID: XP_025819879.1); defensins from oat (*Avena sativa*) (GenBank ID: AST11412.1); gamma-zeathionin-2 from maize (*Zea mays*) (UniProt/SwissProt ID: P81009); and the small protein inhibitor of insect alpha-amylases 1 (SI alpha-1) from sorghum (*Sorghum bicolor*) (UniProtKB/Swiss-Prot: P21923.2). One more peptide from fraction II—Ee-BBTI—shared homology with the Bowman–Birk wound-induced proteinase inhibitor, WIP1-like, from panic grass (*P. virgatum*) (GenBank ID: XP_039813878.1).

**Figure 4:**
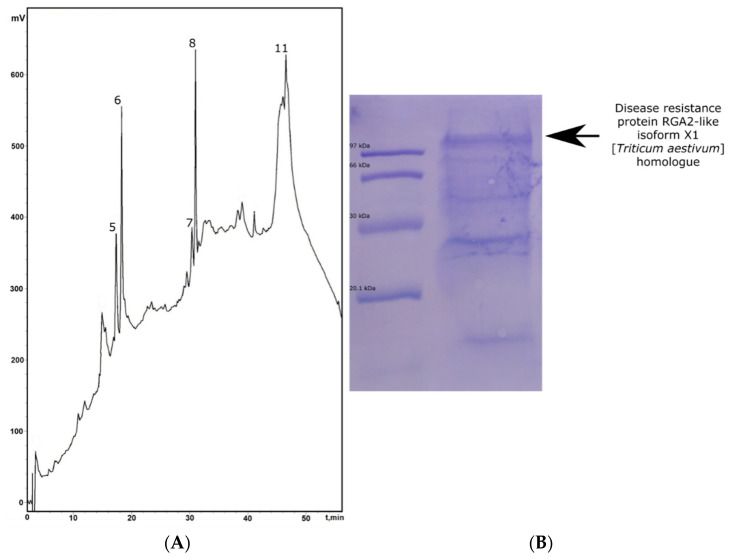
Isolation of the Ee-P1 protein (indicated as peak 11). (**A**)—Analytical RP-HPLC of fraction II after affinity chromatography; (**B**)—Immobilon SQ PVDF membrane after electro-blotting of the subfraction Ee-P1. Molecular weight markers (left track) are presented in a top-down manner: phosphorylase B (97 kDa), BSA (66 kDa), ovalbumin (45 kDa), carbonic anhydrase (30 kDa), and soybean trypsin inhibitor (20.1 kDa). The target protein is indicated by a black arrow and signed.

Additionally, a hydrophobic subfraction (Ee-P1) was detected in fraction II. It was analyzed using SDS PAGE, followed by electro-blotting (Figure 4).

The electropherogram revealed several proteins in this fraction that significantly differ in molecular weight (40 kDa and higher). N-terminal sequencing of the band cutout with mobility between the markers of 14.4–30.0 kDa identified it as belonging to the group of cereal avenin-like storage proteins (avenin-3-like (*Aegilops tauschii* subsp. *strangulata*), GenBank ID: XP_020180381.1). Meanwhile, structural analysis of the most prominent band, named Ee-P1 (with a molecular mass of over 100 kDa), allowed us to identify this protein as a homologue of the disease resistance protein RGA2-like isoform X1 from wheat (*Triticum aestivum*) (GenBank ID: XP_044373989.1).

Typically, such molecules belong to inducible PR proteins, whose gene expression provides a complex immune response of cereals to fungal infections [52,53,54]. Notably, the Ee-P1 analog with a length of over 100 amino acid residues exists in wheatgrass spikelets in a truncated form, lacking the N-terminal region of the polypeptide chain. This partial degradation may be caused by limited proteolysis [55,56]. These proteins were not identified in subfractions Ee100-35B and Ee500-65B. Therefore, they might be located in fractions that did not demonstrate strong antifungal activity.

## 3. Discussion

Proteins and peptides play a remarkable role in plant defense. The most valuable plant organ in terms of population is seed. At the same time, it is the most vulnerable at the stage of germination. This factor might be a cause of high protein and peptide accumulation in the seed endosperm. In this study, we adopted an approach to characterizing the protein extract from seeds that would allow us to assess the contribution of individual components to the constitutive response of a plant to simulated stresses.

At the current stage, we focused on identification of the key molecules that provide the target activity, their structural identification, and an assessment of the action in the complex. This proteomic approach may become widely applicable in the future, particularly with the intensive development of genomic and transcriptomic profiling methods [57,58].

We chose wheatgrass (*E. elongata*), which is a wild plant with increased resistance to salinity and fungal infections; thus, it is an excellent candidate for both traditional and accelerated breeding of some cultivated cereals, such as wheat (*T. aestivum*) [29]. Additionally, with the selected object, the possible relationship between gene transcription, the presence of defense polypeptides of certain structural types, and the manifested forms of resistance can be revealed. Previously determined correlations were shown for wild [6,48,59] and cultivated cereals [41,59,60], as well as some relatives of the cultural forms [61,62,63].

We obtained a total protein and peptide extract from *E. elongata* spikelets. The tests on antimicrobial activity showed that the obtained extract disturbed the growth of fungi from the genus *Fasarium*. This is consistent with *E. elongata*’s increased resistance to fungal diseases. The extract also inhibited disease development in wheat with an artificial background. Another remarkable result represented here is the protection activity of the *E. elongata* extract on yeast cells. Plant–microbe interactions play a vital role in the sustaining of the ecosystem [64]. Fungal communities become sensitive and simpler under high salinity [65]. The yeast model was chosen as it is a well-established test system. The *E. elongata* protein extract preincubation increased cell survival. This primary result brings to mind the application of peptide molecules as regulators of the interactions in microbial communities in agriculture. Nevertheless, this phenomenon requires detailed research.

It was found that biomaterials contain many high-molecular-weight compounds, including, presumably, the water-soluble basic storage proteins. This is typical for cultivated forms and most of their relatives [66], but it has become an obstacle to the detailed characterization of the extract. Nevertheless, defense polypeptides of defensins, lipid-transfer proteins, and hydrolase inhibitor families were found. There are several examples of the successful isolation of individual AMPs from cereal grain [67,68], but in most cases, their greatest diversity can be detected using high-throughput transcriptomic sequencing [6,7,69,70], as well as their prediction [71]. It is worth noting that, despite the presence of gene-coded AMPs, the protein extract inevitably contains some short peptides, which are products of limited proteolysis of functional proteins, particularly chloroplast proteins. They are able to accumulate in grain during maturation. These peptide fragments are known to reveal a diversity of biological functions [72], including regulatory functions [73].

Ee-LTP1-2 and Ee-D1-6 have been identified and partially sequenced. Representatives of these structural families are known to be involved in the plant’s response to both biotic and abiotic stresses [74,75]. Based on this, we can speculate that these defense proteins are involved in the tolerance to a complex of abiotic stresses, particularly salinity. The revealing of antifungal molecules in the extract is consistent with its antifungal activity. At the same time, the trypsin inhibitor and bifunctional trypsin/alpha-amylase inhibitors found in the extract might participate in the resistance to diseases and the pest *E. elongata*. However, it is important to note that stress tolerance involves a combination of the numerous molecular factors, such as the regulation of transcription and translation, that form a part of induced plant immunity.

Cereal seeds contain a significant number of so-called disease resistance proteins. Many of them have been predicted from genomic and transcriptomic data. Some of these molecules can be classified as PR proteins according to their primary structure homology [76]. The specific functions of such proteins are poorly understood and require careful consideration. In some cases, their isolation in a native form is a challenge due to their high molecular weight and representation by several isoforms. Here, we represent the isolation of one such protein called Ee-P1.

This study contributes to the conception of the agricultural application of AMP properties. In general, there are two strategies for AMP use. The first involves the isolation of pure substances from biomaterials or chemical synthesis for pure preparation. The second involves the application of AMP properties without the isolation and purification of any substances, but it suggests the use of transformed organisms and novel agricultural methods. There is no way to obtain enough extract with the standard properties and compound ratios [77]. Thus, the obtained results support the second strategy in terms of the directed breeding and development of transformed plants.

Under natural conditions, protective molecules act simultaneously. Therefore, we attempted here to have a look through the complex action of proteins in three in vitro models without focusing on single molecules. Moreover, these results suggest that there is a protective action of plant proteins on the microbiome under stress conditions; this action allows plant immunity at the ecosystem level. The complex analysis of the naturally occurring defense peptides described in this study is the first example of an examination of the contribution of single molecules to both the biotic and the abiotic stress response.

## 4. Conclusions

Finally, we obtained a protein and peptide extract from wheatgrass (*E. elongata*) and studied its biological activity. This extract inhibits phytopathogenic fungi from the genus *Fusarium* and suppresses the development of disease symptoms in wheat grain (*T. aestivum*). We also found that this extract could defend yeast culture under the action of hyper-salinity stress. Further structural analysis revealed the presence of several AMPs and hydrolase inhibitors involved in the immune response of this cereal to biotic stress. For the first time, we confirmed the presence of a disease resistance protein in a wild cereal, which could be involved in the complex protection of a plant from stresses of various origins.

## 5. Materials and Methods

### 5.1. Biological Material

#### 5.1.1. Plant

Spikelets of *Elytrigia elongata* ((Host.) Nevski) (syn. *Agropyron elongatum* (Host) Beauv., *Elymus elongatus* (Host) Greuter comb. superfl., *E. elongatus* (Host) Runemark, *Elymus elongatus* subsp. *ponticus* (Podp.) Melderis, *Elytrigia prokudinii* Druleva) were collected in August 2013 from the shore of the Black Sea (Nakhimovsky district, the northern part of Sevastopol, Republic of Crimea). The spikelets were collected in the area with the following GPS coordinates: from 44.635080 N to 44.636886 N and from 33.515239 to 33.517502 E.

#### 5.1.2. Microorganisms

The following cultures were used: *Bipolaris sorokiniana* strain VKM F-1446, *Fusarium graminearum* strain VKM F-1668, *Fusarium culmorum* strain VKM F-844, and *Aspergillus niger* strain VKM F-33. They were purchased from the All-Russian Collection of Microorganisms, IBPM RAS (Pushchino, Russia). The *Saccaromyces cerevisiae* strain VKPM Y-1200 was selected from the All-Russian Collection of Industrial Microorganisms “GOSNII Genetika” (Moscow, Russia).

### 5.2. Preparation of Total Protein Extract from E. elongata Spikelets

To obtain the protein extract (PE) from the biomaterial, the principal scheme was applied [78]. Firstly, the dried spikelets (100 g) were crushed with a coffee mill (Bosch, Gerlingen, Germany); then, flour was further extracted using 10% acetic acid (Khimmmed, Moscow, Russia) in water (1:10, *w*/*v*) for 1.5 h at room temperature with intensive stirring. After centrifugation (7000 rpm, 10 min, 4 °C), the supernatant was collected and partially evaporated, and the polypeptides were precipitated using cooled acetone (Khimmmed, Russia) (1:10, *v/v*) overnight. The next morning, a pellet was collected and air-dried at room temperature for 24 h. At the final stage of fractionation, the dried pellet was dissolved in 0.1% trifluoroacetic acid (TFA) and desalted by RP-HPLC using an Aquapore C_8_ (10 × 100 mm, 7 µm, 300 Å) (Applied Biosystems, Waltham, MA, USA). An eluate was partially evaporated using the SpeedVac Vacuum Concentrator (Labconco, Kansas City, MO, USA) and lyophilized (Free-Zone, Labconco, Kansas City, MO, USA).

### 5.3. Medium-Pressure Affinity Chromatography

The lyophilized PE was dissolved in 10 mM tris-HCl (pH 7.2) and applied to a Heparin HiTrap Sepharose column (GE Healthcare, Chicago, IL, USA). After the release of all the unbound components from the column, the separation was carried out in a stepwise gradient of an increasing concentration of NaCl in 10 mM tris-HCl (pH 7.2) up to 100 and 500 mM, respectively. The flow rate of the mobile phase was 1.5 mL/min. Detection of absorbance was monitored at a wavelength of 280 nm.

### 5.4. Stepwise RP-HPLC

The fractions collected after medium-pressure affinity chromatography were further separated by semi-preparative RP-HPLC using the Aquapore C_8_ (10 × 250 mm, 7 µm, 300 Å) (Applied Biosystems, Waltham, MA, USA), equilibrated with 15% solvent B (80% MeCN in 0.1 TFA). After elution of the subfraction, the next two steps were serially performed (35% B and 65% B), and all the eluates were collected manually. All of the total subfractions were evaporated and lyophilized.

### 5.5. Analytical RP-HPLC

The fractions obtained after stepwise HPLC were analyzed by gradient RP-HPLC using the XBridge BEH C_18_ (4.0 × 250 mm, 3.6 µm, 300 Å) column (Waters, Drinagh, Ireland) in a linear gradient of solvent B (80% MeCN in 0.1 TFA) relative to solvent A (0.1 TFA) (5–50% for 40 min, 50–75% for 10 min, 75–90% for 1 min, and isocratic 90% for 10 min). The flow rate of the mobile phase was 1.0 mL/min, and detection was performed at 214 nm. The fractions obtained after affinity chromatography were analyzed by gradient RP-HPLC using the Aeris C_4_ (4.0 × 250 mm, 3.6 µm, 200 Å) column (Phenomenex, Torrance, CA, USA) in a linear gradient of solvent B (80% MeCN in 0.1% TFA) relative to solvent A (0.1 TFA) (5–50% for 40 min, 50–75% for 10 min, 75–90% for 1 min, and isocratic 90% for 10 min). The flow rate of the mobile phase was 1.0 mL/min, and detection was performed at 214 nm.

### 5.6. SDS-PAGE and Electro-Blotting

Electrophoresis was performed under reducing conditions in 10% PAAG according to the Laemmli method. A mixture of purified standard proteins was applied as a marker: phosphorylase B (97 kDa), BSA (66 kDa), ovalbumin (45 kDa), carbonic anhydrase (30 kDa), and soybean trypsin inhibitor (20.1 kDa). Electrophoresis was conducted at a current of 10 mA until complete insertion of bromophenol blue into the gel, and then at 15 mA. After the end of the process (1.5–2 h), the gel was transferred onto an Immobilone PVDF membrane (Millipore, Burlington, MA, USA). After electro-blotting, the membrane was stained with 0.1% Coomassie Brilliant Blue R-250 solution in 50% aqueous methanol for 3–5 min, then decolorized in 50% aqueous methanol and washed with MQ water.

### 5.7. MALDI-TOF/TOF MS

MALDI MS analysis was performed using an Autospeed MALDI-TOF instrument (Bruker Daltonics, Bremen, Germany) in positive ion mode. 2,5-dihydroxybenzoic acid (DHB) (Sigma-Aldrich, St. Louis, MO, USA) at a concentration of 20 mg/mL of 80% MeCN in water was used as a matrix. The analyzed sample solution (0.6 μL) was mixed with an equivalent volume of the matrix and applied to the target. Mass spectra were analyzed using the mMass—Open Source Mass Spectrometry Tool v. 5.5.0 (http://www.mmass.org/).

### 5.8. N-Terminal Sequencing

Partial primary structure analysis of the individual peptides was carried out by Edman automatic stepwise degradation using a protein and peptide sequencer PPSQ-33A (Shimadzu Corp., Kyoto, Japan) according to the manufacturer’s protocol. For the analysis, approximately 500–700 pmol of polypeptide purified by analytical RP-HPLC was taken and dissolved in 30μL of 50% acetonitrile with the addition of 0.1% TFA. The proteins were analyzed after the cutting of the Immobilone PVDF membrane and transferred to a reactor. The identification of the obtained PTH derivatives of the amino acids with respect to the calibration profile was performed using the LabSolution PPSQ software v. 1.0 (Shimadzu Corp., Kyoto, Japan).

### 5.9. Antifungal Activity In Vitro

Antifungal assays were determined by disc-diffusion assay using sterile paper filter disks moistened with a solution of the tested substance and air-dried under sterile conditions. The PPE was dissolved in a range of active concentrations of 0.25–2.0 mg/mL in 25% EtOH. The sensitivity of the test organism was controlled by standard discs with amphotericin B at a concentration of 40 µg/disc (Saint Petersburg Pasteur Institute, Saint Petersburg, Russia).

### 5.10. Treatment of Wheat Grain

To check the contact action effectiveness of the wheatgrass protein extract, the development of the plant pathogenic fungus *Fusarium gramimearum* suppression on wheat grain was chosen. The extract was dried and dissolved in 70% aqueous ethanol. Three concentrations were applied: 1, 2, and 4 mg/mL. Aqueous ethanol (70%) was applied as a control. The wheat grain (*Triticum aestivum*) was soaked in the extract or ethanol solutions for an hour. Subsequently, the grain was removed from the solutions, air-dried, and placed in Petri dishes on filter paper moistened with tap water. Afterwards, a suspension of mycelium and conidia of *F. graminearum* with a titer of approximately 20,000 CFU/mL was applied to the surface of each grain, followed by incubation in the dark at a constant temperature of 25 °C. The pathogen development degree on the surface of the wheat grain was assessed by the relative intensity of conidial sporulation on the substrate after 72 h and evaluated as follows: from “−” to “++++”, with “++++” being complete inhibition of the disease symptoms; “+++” is disease development of less than 10%; “++” is disease development of less than 20%; “+” is disease development of less than 40%; and “−” is disease development of 40% (the absence of inhibition).

### 5.11. Protection Assays In Vitro

Bile salts (sodium cholate and deoxycholate) (BS) were used as stress factors. In the control variants, cell suspensions of the test strains in physiological saline (1 mL) were incubated for 30 min at 33 °C with an equal volume (1 mL) of a solution of the bile salts (2 g/L, pH 7.0). In the experimental variants, 1 mL of the PE solution (2 mg/mL) was added to 1 mL of the cell suspension of the test strains 10 min before the addition of the BS solution to determine the protective effect. Afterwards, an equal volume (2 mL) of the BS solution was added and incubated as described above. Then, ten-fold dilutions of the sample were prepared, and seeding was carried out in Petri dishes with culture medium to determine the number of surviving cells (CFU/mL). For inoculation, a micro-method was used involving the application of an aliquot of microbial suspensions with a volume of 5 μL to an agar medium, with six-fold repetitions for each dilution. The effectiveness of the protective or reactivating action of the extracellular metabolites of *S. cerevisiae* was determined as a fission index (ID) and estimated using the ratio of the number of CFUs in the cell suspensions incubated with PE after exposure to a stress-protective factor to the number of CFUs in a suspension subjected only to the stress effect of BS [79].

### 5.12. Statistical Analysis

In the presence of a normal distribution, Student’s *t*-test was used. The data are presented as the mean and standard deviation (mean ± SD). The differences were considered significant at a *p*-value ≤ 0.05.

## Figures and Tables

**Table 1:** In vitro antifungal activity of EePE from wheatgrass (*E. elongata*) spikelets *. Antifungal activity is represented as the diameter of growth suppression zones (mm) *.

Fungal Species	Concentration of PE, mg/mL				Amphotericin B
	0.25	0.5	1.0	2.0	
*B. sorokiniana*	-	-	-	-	+(30 ± 2)
*F. graminearum*	-	-	+(14 ± 1)	+(20 ± 2)	+(34 ± 4)
*F. culmorum*	-	-	+(15 ± 2)	+(22 ± 3)	+(28 ± 1)
*A. niger*	-	-	-	-	+(26 ± 2)

* EePE solutions were applied on paper discs, which were placed on solid media seeded with fungal conidia. Activity of the extract was detected according to the growth suppression zone formation. Zones formed were measured manually.

**Table 2:** Partial structure identification of the individual polypeptides isolated from the wheatgrass (*E. elongata*) protein extract, performed using automated N-terminal Edman sequencing.

Peak	Average Molecular Mass Measured, kDa	Peptide Title	N-Terminal Amino Acid Sequence	Annotation
1	11.865	Ee-BFTI	^1^SSPSTCVAGEAIPGRP^16^	Bifunctional trypsin/alpha-amylase inhibitor from *Poaceae*
2	5.612	Ee-D5	^1^KECKTGSAGYKGPC^14^	Plant defensin
3	10.038	Ee-LTP1	^1^ISCLPYVDGQGKSP14	Lipid-transfer protein (the 9 kDa subfamily)
4	9.895	Ee-LTP2	^1^IICLPYVDGQTKSP14	Lipid-transfer protein (the 9 kDa subfamily)
5	5.938	Ee-D1	^1^KICRQKSAGVIGPC^14^	Plant defensin
6	5.920	Ee-D2	^1^KICRNRSAGFRGPC^14^	Plant defensin
7	5.034	Ee-D3	^1^KVCTGKGQDHSFPC^14^	Plant defensin
8	5.019	Ee-D4	^1^KVCTGKSQDHSFPC^14^	Plant defensin
9	5.728	Ee-D6	^1^KFCRTRSAGYRGPC^14^	Plant defensin
10	6.903	Ee-BBTI	^1^KKKGCCNNCQSWSG^14^	Bowman–Birk trypsin inhibitor
11	>100.0	Ee-P1	^1^LETITSQHRSFS12	Disease resistance protein RGA2-like isoform X1 [*Triticum aestivum*] homologue

## Data Availability

The data presented in this study are available on request from the corresponding authors.
